# Editorial: Advancing livestock health: innovative vaccine strategies to combat antimicrobial resistance

**DOI:** 10.3389/fimmu.2026.1833018

**Published:** 2026-04-07

**Authors:** Md Bashir Uddin, Chamith Hewawaduge

**Affiliations:** 1 Department of Medicine, Sylhet Agricultural University, Sylhet, Bangladesh; 2 Sri Lanka Institute of Biotechnology, Homagama, Sri Lanka

**Keywords:** animal health, antimicrobial resistance (AMR), disease prevention, livestock vaccines, one health, sustainable production

Antimicrobial resistance (AMR) has emerged as one of the most critical global health challenges of the 21st century, threatening livestock productivity, food security, and public health. The extensive and often indiscriminate use of antimicrobials in livestock has accelerated the emergence of resistant pathogens, reducing therapeutic efficacy and complicating disease management across production systems. Vaccination represents one of the most promising preventive interventions capable of mitigating infection pressure while preserving antimicrobial efficacy. Recognizing the urgent need for transformative solutions, this Research Topic “*Advancing Livestock Health: Innovative Vaccine Strategies to Combat Antimicrobial Resistance*” brings together seven contributions that collectively highlight the growing role of vaccination as a central component of antimicrobial stewardship in livestock system. These studies span experimental research, vaccine platform development, and providing a comprehensive overview of emerging strategies that can reduce antimicrobial reliance while strengthening animal health within a One Health framework.

A central theme across the Research Topic is the strategic targeting of bacterial pathogens that drive substantial antimicrobial usage in food-producing animals. Rossi et al., investigates humoral and cellular immune responses in cattle following vaccination and challenge with *Clostridium chauvoei*, the causative agent of blackleg. By evaluating both antibody-mediated immunity and T-cell responses, the authors demonstrate that effective protection involves coordinated adaptive immune mechanisms. These findings highlight the importance of identifying immune correlates of protection when evaluating vaccine efficacy and optimizing vaccination strategies for clostridial diseases in cattle.

Razavi et al. examines peptide-based vaccines as a promising therapeutic and preventive strategy against antibiotic-resistant bacterial infections. Peptide vaccines offer several advantages, including high specificity, safety, and the potential for rational design targeting conserved antigenic determinants. The review highlights advances in peptide vaccine development, including epitope mapping, structural optimization, and delivery strategies that enhance immunogenicity. Such approaches may enable the development of vaccines capable of targeting antimicrobial-resistant pathogens while minimizing off-target immune responses.

Singh et al. explored functional antibody responses by investigating influenza-specific antibody-mediated and complement-dependent cellular cytotoxicity in vaccinated and infected pigs. The findings emphasize that protective immunity extends beyond neutralizing antibodies and involves additional effector functions such as antibody-dependent cellular cytotoxicity (ADCC) and complement activation. These mechanisms contribute to viral clearance and enhanced immune protection, underscoring the importance of evaluating functional antibody responses when assessing vaccine-induced immunity in swine populations.

The application of immunoinformatics and rational antigen design represents another key advance highlighted in this Research Topic. Shah et al., reports the identification of novel therapeutic targets and the construction of a chimeric vaccine candidate against antibiotic-resistant *Yersinia enterocolitica*. Using computational screening and epitope prediction approaches, the authors designed a multi-antigen construct capable of eliciting both humoral and cellular immune responses. This work illustrates the potential of bioinformatics-driven vaccine development to accelerate antigen discovery and generate targeted vaccine candidates against emerging resistant pathogens.

Optimizing cellular immunity through co-vaccination strategies is also explored by Sterle et al., evaluating the effects of co-administration of RB51 and BCG vaccines in cattle. The authors demonstrate that co-vaccination enhances vaccine-specific CD4^+^ T-cell responses, suggesting a potential strategy to improve protective immunity against intracellular bacterial pathogens. These findings provide important insights into how vaccine combinations may modulate immune responses and enhance protective efficacy in livestock.

Zhang et al., evaluated a multi-epitope vaccine candidate targeting *Pasteurella multocida*, a pathogen responsible for significant respiratory disease in livestock. Using a rationally designed multi-epitope construct, the study demonstrates promising immunogenicity and protective efficacy in a mouse model. Multi-epitope vaccine platforms offer the advantage of incorporating multiple antigenic determinants, thereby broadening immune coverage while reducing the risk of antigenic escape.

Complementing these experimental studies, a comprehensive review by Tesfaye et al., examines advances in vaccine development strategies for *Actinobacillus pleuropneumoniae*, a major respiratory pathogen affecting swine production. The review summarizes current progress in subunit vaccines, recombinant proteins, live attenuated platforms, and novel delivery systems. By highlighting emerging technologies and immunological insights, the article provides a roadmap for developing next-generation vaccines capable of controlling respiratory diseases in swine while reducing antimicrobial interventions.

Collectively, these contributions highlight several key advances: identification of immune correlates of protection in cattle and swine; development of peptide-based and multi-epitope vaccines; rational antigen design using immunoinformatics; exploration of functional antibody mechanisms; and co-vaccination strategies to enhance cellular immunity. These studies underscore that vaccination is not only a disease control tool but a strategic intervention to mitigate AMR.

Effective vaccination reduces infection incidence and severity, limiting both therapeutic and prophylactic antimicrobial use. Vaccines also decrease pathogen shedding and environmental contamination, disrupting transmission within and between farms. By lowering infection pressure, vaccination diminishes the selection of resistant strains, contributing to broader One Health objectives. However, challenges remain, including pathogen diversity, antigenic variation, cold-chain requirements, and regulatory harmonization. Translational research should prioritize durable immunity, cross-strain protection, scalable manufacturing, and practical deployment in resource-limited settings.

In conclusion, this Research Topic emphasizes vaccination as a transformative pillar for sustainable livestock health and AMR mitigation. The studies presented illustrate how integrating immunology, molecular biology, and computational approaches can accelerate vaccine innovation, reduce antimicrobial reliance, and safeguard food systems. Strengthening vaccine-driven prevention strategies is essential to protect animal health, promote sustainable production, and preserve antimicrobial efficacy within a One Health framework.

We thank all contributing authors, reviewers, and the editorial team for their commitment to scientific rigor and collaborative progress. We hope this Research Topic stimulates further research, encourages interdisciplinary partnerships, and advances global efforts to control AMR through vaccination.

